# Book Reviews

**Published:** 1900-03

**Authors:** 


### BOOK REVIEWS, PAMPHLETS, EXCHANGES.

### BOOK REVIEWS.

[Contributions solicited for review.]

THE ESSENTIALS OF PHYSICAL DIAGNOSIS OF THE THORAX. By Arthur M. Cowan, M.D., Chicago. Third edition. Revised and enlarged. W. B. Saunders, Philadelphia, Pa. 1899.

In this small volume are set forth the principles of physical diagnosis of the thorax under the heads of topography of the chest, methods of physical diagnosis, signs in diseases of the chest, signs in diseases of the heart, signs in diseases of the aorta. This covers the ground and is arranged in an instructive manner. Every one appreciates the importance of physical diagnosis and the advantage given him by a good knowledge of the subject. This book will give him that knowledge.

THE SURGICAL DISEASES OF THE GENITO-URINARY TRACT, VENEREAL AND SEXUAL DISEASES. A Text-book for Students and Practitioners. By G. Frank Lydston, M.D., Professor of the Surgical Diseases of the Genito-Urinary Organs and Syphilology in the Medical Department of the State University of Illinois; Professor of Criminal Anthropology in the Kent College of Law; Surgeon-Chief of the Genito-Urinary Department of the West-Side Dispensary; Fellow of the Chicago Academy of Medicine; Fellow of the American Academy of Political and Social Science; Delegate from the United States to the International Congress for the Prevention of Syphilis and the Venereal Diseases, held at Brussels, Belgium, September 5, 1899, etc. Illustrated with 233 Engravings. $6\frac{1}{2}$x$9\frac{3}{4}$ inches. Pages xvi–1024. Extra Cloth, $5.00 net. Sheep or Half-russia, $5.75 net. The F. A. Davis Co., Publishers, 1914-16 Cherry St., Philadelphia.

Under this comprehensive title Dr. Lydston has issued a volume which is in every way a credit to this well-known writer and a valuable edition to the literature of the subject. Somewhat more space than is usual in a work of this character is devoted to affections of the kidney and the operative procedures thereon. This might have been considered as more properly belonging to the field of general surgery. The several types of defective development of the sexual organs are discussed and generally illustrated. Sexual perversion is rather extensively dealt with. The book is extremely well written. The author has an attractive way of stating things which goes far toward sustaining the reader's interest. Lydston will probably rank among the classics.

DIAGNOSIS OF THE URINE, OR THE PRACTICAL EXAMINATION OF URINE WITH SPECIAL REFERENCE TO DIAGNOSIS. By Alfred Memminger, M.D., Professor of Chemistry, Urinology and Hygiene in the Medical College of the State of South Carolina. 2d edition, enlarged and revised, with illustrations. Philadelphia. P. Blakiston's Son & Co. 1899.

This represents a thorough revision of the now exhausted first edition of this useful little book. A chapter has been added on the differential diagnosis of chronic Bright's disease, or a classification of normal absolute, the absolute and the relative absolute of solids and urea. The student and practitioner will find much useful information in the book, which is well worth the modest price of $1.00.

---

THE URINE AND CLINICAL EXAMINATION OF THE GASTRIC CONTENTS, THE COMMON POISONS AND MILK. By J. W. Holland, Professor Medical Chemistry and Toxicology Jefferson Medical College of Philadelphia. 41 illustrations. 6th edition, revised and enlarged. Philadelphia. P. Blakiston's Son & Co. 1899.

This book is a laboratory guide. It is well illustrated, clear and brief, and is intended to slip in the pocket like a note-book. The pages are printed on one side, leaving the other side blank for note-taking. This arrangement will appeal to the student, as will also the price, which is $1.00, not much more than the cost of a note-book.

